# The Iceman’s microbiome: unveiling millennia of microbial diversity and continuity

**DOI:** 10.1186/s40168-026-02417-6

**Published:** 2026-06-03

**Authors:** Mohamed S. Sarhan, Marco Samadelli, Albert Zink, Frank Maixner

**Affiliations:** 1 Eurac Research – Institute for Mummy Studies, Bolzano, 39100 Italy; 2 Eurac Research – Institute for Biomedicine, Bolzano, 39100 Italy; 3 Department CIBIO, University of Trento, Trento, 38123 Italy

**Keywords:** Iceman, Ancient microbiome, Frozen mummy conservation, Psychrophilic yeasts, Ancient DNA, Iceman, Ötzi, Mummies

## Abstract

**Background:**

The Iceman mummy, a 5300-year-old natural alpine glacier mummy, provides a unique opportunity to study ancient microbial ecosystems. However, disentangling the mummy’s endogenous microbiome from modern environmental contaminants introduced during three decades of conservation remains a significant challenge.

**Results:**

By integrating culture-dependent and culture-independent approaches, including amplicon sequencing, shotgun metagenomics and de novo metagenomic assembly, as well as isolate-level genomics, we performed a comprehensive characterization of the Iceman’s microbial landscape. We identified three distinct microbial drivers: endogenous post-mortem succession, ancient glacier-derived relicts, and modern anthropogenic introduction. Metagenomic analysis of internal tissues revealed anaerobic bacteria, including ancient gut taxa, including such as *Romboutsia hominis*, *Clostridium moniliforme*, *Eubacterium* sp., *Ruminococcus bromii*, *Kineothrix* sp., *Treponema succinifaciens*, *Enterousia* sp., and *Huintestinicola butyrica*. These taxa, characterized by ancient DNA (aDNA) damage profiles (C to T deamination frequency), show high similarity to ancestral, non-Westernized human gut communities, providing a rare baseline for Copper Age intestinal ecosystems. Conversely, we identified a shift in the external mycobiome, marked by the recent proliferation of psychrophilic yeasts, including *Glaciozyma watsonii*, *Mrakia robertii*, *Phenoliferia glacialis*, and *Goffeauzyma* sp. While internal bacterial communities remained stable, these external yeast populations showed increased relative abundance and reduced DNA damage signatures between 2010 and 2019, indicating active, modern colonization. Furthermore, strain-level analysis of *Pseudomonas* sp. 5C2 confirmed that specific environmental strains have successfully colonized the mummy, persisting across multiple tissue sites with minimal genetic divergence.

**Conclusions:**

Our study demonstrates that the Iceman is not a static relic but a dynamic biological interface. The coexistence of ancient, endogenous gut microbes and modern, psychrophilic colonizers highlights the potential for ongoing microbial activity even at sub-zero temperatures. These findings underscore that maintaining strict environmental parameters is essential to prevent these specialized microbial communities from transitioning from latent persistence to active microorganisms.

Video Abstract

**Supplementary Information:**

The online version contains supplementary material available at 10.1186/s40168-026-02417-6.

## Background

More than 5300 years, the Iceman’s body was preserved in low-temperature surroundings of the glacier, which protected the mummy from further degradation, e.g., microbial decomposition. In 1991, the mummy was discovered in the Ötztal Alps close to the nowadays Austrian Italian borders. After his discovery in 1991, the mummy was treated with a phenol-containing solution to avoid fungal growth [1, 2], and then the responsible scientists realized the necessity to store the body in a frozen state to avoid further microbial up growth. Currently, the Iceman mummy is preserved in a refrigeration chamber at a constant temperature of −6 °C and a relative humidity of 99% at the South Tyrolean Museum of Archeology in Bolzano, Italy [3]. The current preservation parameters most closely correspond to the conditions inside the glacier where the mummy was found, except for an elevated oxygen concentration (about 30% higher) due to differences in altitude.

Thus, the current storage conditions exclude already the up growth of most microorganisms involved in decomposition, which have higher optimal growth temperatures and are often strict anaerobic. Nevertheless, the current storage temperature of the Iceman (−6 °C) still represents an ecological micro-environment for cold-adapted microorganisms, i.e., psychrophilic or psychrotolerant microbes.

Despite decades of careful monitoring, a critical question remains unresolved: Do the current conservation conditions—specifically −6 °C, 99% relative humidity, and elevated oxygen—truly prevent microbial growth, or do they instead permit the slow metabolic activity of cold-adapted microorganisms that could be involved in biodeterioration over time? While prior studies have detected microbial DNA within the Iceman’s tissues [4, 5], they have not distinguished between dormant, dead, or metabolically active cells—nor have they evaluated whether resident microbes retain the capacity to grow under the museum’s storage regime. This represents a significant knowledge gap: without evidence of microbial viability, activity, or functional potential under in situ conditions, we cannot accurately assess the long-term biodeterioration risk. Cultivation-based surveillance alone is insufficient, as it overlooks the vast majority of unculturable taxa and provides no insight into community structure or metabolic capabilities.

Furthermore, the characterization of the modern microbial community is not only a prerequisite for conservation but also a critical step for understanding the ancient microbiome of a Copper Age individual. By precisely defining the modern environmental influx and the patterns of recent colonization, we can more effectively disentangle the authentic, ancient signals from modern noise. This approach allows for the rigorous identification and reconstruction of the endogenous gut microbiota—remnants of the Iceman’s original intestinal ecosystem that have remained preserved for more than 5000 years [6, 7].

In this study, we conducted a comprehensive microbiological assessment combining amplicon-based profiling, shotgun metagenomics, and targeted cultivation, enabling us to characterize the taxonomic composition, functional potential, and cultivable fraction of microbial communities associated with the Iceman and his conservation chamber. Our aim is to determine whether active or potentially active microorganisms persist under current storage conditions—and if so, to evaluate their role in the mummy’s preservation.

## Results

### Sampling strategy and compositional diversity of the Iceman microbiome

**Fig. 1:**
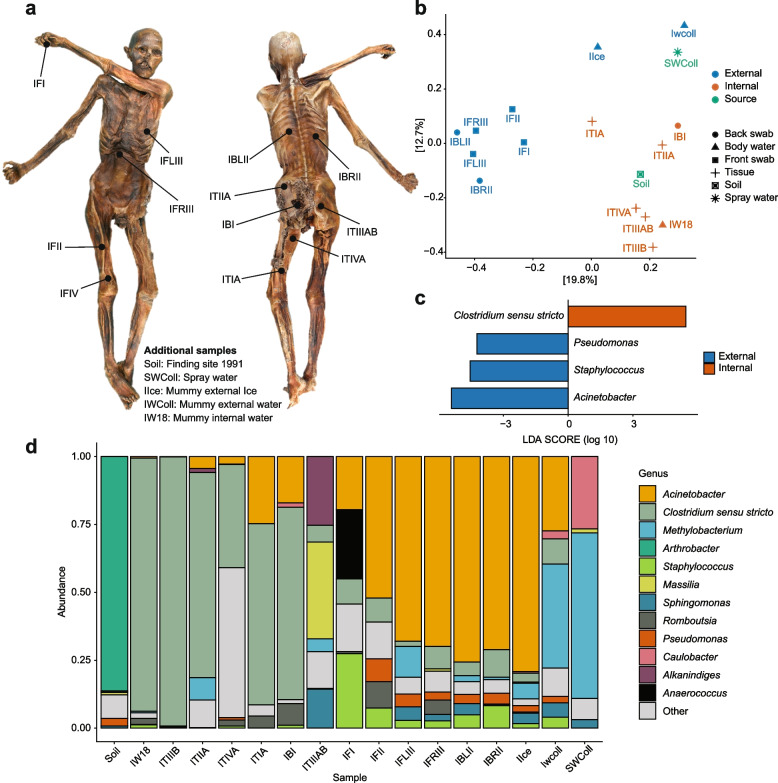
Culture-independent analysis of the Iceman microbiome. **a** Anatomical sampling sites on the Iceman’s body (front and back views). Swab samples were collected from specific locations (labeled IFI–ITIVA). Additional samples were included in the analysis: Soil, soil collected from the finding site in 1991; SWColl, spray water that is being used for humidity regulation of the mummy; IIce, the external ice blocks covering the mummy before defrosting; IWColl, the thawed water during the defrosting process; IW18, the thawed internal water of the mummy. Further details of the samples are provided in Table S1. **b** Principal coordinates analysis (PCoA) at the 16S rRNA ASV-level, based on weighted Bray-Curtis distances. Different colors refer to sample depths: External, samples that were in contact with the outer body parts; Internal, internal swabs, tissue samples, and internal water; and source, potential source samples (i.e., soil and spray water). **c** Effect of sample group (i.e., internal or external) on the Iceman microbiome composition at genus level using linear discriminant analysis (LDA) with effect size measurements (LEfSe). **d** Relative abundance of the top 12 bacterial genera representing > 95% of whole community

To evaluate the current microbiological status of the Iceman and distinguish between ancient signatures and modern environmental contaminations, we implemented a comprehensive sampling protocol covering the mummy, its immediate conservation environment, and the original finding site controls (Fig. 1a, Fig. S1). Initially, to monitor the possibility of modern contamination, air-borne spore sampling was conducted across a spatial gradient within the South Tyrolean Museum of Archaeology, encompassing the main conservation chamber and adjacent laboratory facilities (Fig. S1). Subsequently, under strict aseptic conditions, the mummy was defrosted at 4 °C; during this process, external ice blocks (IIce) and water samples were collected (IWColl, Fig. S2). After partial defrosting, we collected 12 anatomical swabs (IFI–ITIVA) as well as skin tissue fragments, including muscle and connective tissue (Fig. S2). At the conclusion of the sampling, internal thawed water (IW18-IW20) was collected. Additionally, original soil from the 1991 finding site and humidity-regulating spray water (SWColl) were included to define potential microbial source tracks (Table S1).

To analyze the microbiome of the collected samples, we employed 16S rRNA gene amplicon analysis (Table S2, S3, Methods). Principal coordinates analysis (PCoA) based on ASV-level distances demonstrated a clear ecological separation based on sample depth (Fig. 1b). A pairwise PERMANOVA based on Bray–Curtis dissimilarities showed that microbial community composition differed significantly between internal and external samples (*R*^2^ = 0.176, *p* = 0.002). External skin swabs and environmental “source” samples, such as soil and spray water, clustered distinctly from internal tissues. This suggests that the internal mummy environment maintains a unique microbial signature that is fundamentally different from the modern conservation facility. Linear discriminant analysis effect size (LEfSe) further identified the taxa driving this clustering, revealing that internal samples were significantly enriched with anaerobic genera, most notably *Clostridium *sensu stricto (Fig. 1c). Conversely, external surfaces were characterized by common environmental and skin-associated bacteria, including *Pseudomonas*, *Staphylococcus*, and *Acinetobacter*.

Taxonomic profiling confirmed that the internal samples (e.g., ITIIIA, ITIVA) were dominated by *Clostridium* spp., which are typically associated with the preservation of mummified remains (Fig. 1d). In contrast, environmental controls like the 1991 soil showed a prevalence of *Arthrobacter* and *Massilia*. Our investigation into the external influence revealed that the mummy’s surface is not merely a reflection of ambient air but is instead directly and dominantly shaped by the spray water used in its conservation. Unexpectedly, the spray water sample was dominated almost exclusively by *Methylobacterium* (61%), *Caulobacter* (26.5%), *Bradyrhizobium* (7.8%), *Sphingomonas* (3%), and *Massilia* (1.6%). Notably, *Methylobacterium* and *Bradyrhizobium* were detected across all sample groups, but their relative abundance followed a distinct spatial gradient, decreasing progressively from the external surfaces toward the internal tissues (Fig. 1d, Table S4).

Interestingly, the “Body Water” (IW18) and certain skin sites exhibited transitional profiles, containing a mixture of environmental taxa and specialized microbes (e.g., *Romboutsia* and *Clostridium*). The high prevalence of spray-water-associated taxa (specifically the *Methylobacterium*-rich signature) on the exterior highlights the impact of past humidification protocols on the mummy’s surface microbiome.

### Cultivation-based analysis of fungal and bacterial isolates

To complement the culture-independent analysis, we performed cultivation experiments on samples from the Iceman’s body and air-borne spores from the surrounding museum environment to identify viable microbial components. Cultivation of air-borne spores from the Iceman conservation chamber (I), laboratory (II), and monitoring office (IV) revealed a broad diversity of fungi, predominantly represented by common environmental and indoor molds such as *Cladosporium* spp., *Penicillium* spp., and *Alternaria* spp. [8]. Within the conservation chamber and laboratory air, we also recovered psychrotolerant species such as *Pseudogymnoascus pannorum* and the bacterium *Massilia brevitalea*, suggesting a selective pressure for cold-adapted microbes within the facility (Table S5).

In contrast to the indoor air, cultivation from the Iceman’s skin (IBI, IBRII) and internal “late water” (IW18, IW20) yielded specialized microbial lineages. Notably, *Staphylococcus* spp. were successfully isolated from the back swab (IBRII) and internal water (IW18-1, IW18-2). These isolates directly correspond to the high relative abundance of *Staphylococcus* observed in the 16S rRNA amplicon profiling of external samples (Fig. 1c, d), suggesting that a portion of the skin-associated microbiome identified metagenomically might remain viable.

**Fig. 2:**
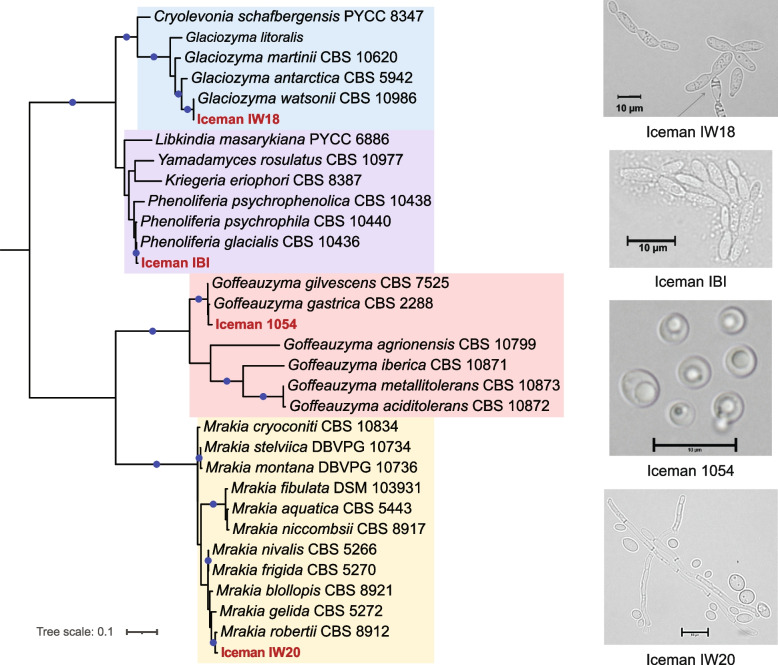
Yeast isolates recovered from Iceman samples**.** On the left side, maximum-likelihood phylogenetic tree based on ITS sequences showing the placement of Iceman yeast isolates (in red) among reference taxa. The clades containing each isolate are highlighted by shaded boxes corresponding to different families and genera. On the right side, morphological diversity of yeast isolates recovered from Iceman samples, observed under light microscopy. Scale bars: 10 µm

Furthermore, the cultivation yielded four distinct yeast strains: *Glaciozyma watsonii* (IW18), *Mrakia robertii* (IW20), *Phenoliferia glacialis* (IBI), and a *Goffeauzyma* species (Iceman 1054). Morphological observation confirmed diverse yeast-like structures, including budding cells and pseudohyphae characteristic of these basidiomycetous yeasts (Fig. 2, right panel). Phylogenetic analysis based on Internal Transcribed Spacer (ITS) sequences and whole-genome phylogenomics placed these isolates within distinct clades of cold-adapted yeasts (Fig. 2, Fig. S3). *Glaciozyma* and *Mrakia* isolates clustered closely with reference taxa from Arctic and Antarctic environments, highlighting their psychrophilic nature.

To provide a higher-resolution taxonomic resolution for the recovered yeasts, we performed whole-genome sequencing (WGS) on the four primary isolates (*Glaciozyma* IW18, *Mrakia* IW20, *Phenoliferia* IBI, and *Goffeauzyma* 1054) (Table S6). This genomic approach allowed for a robust phylogenomic reconstruction by comparing the Iceman isolates against all available NCBI reference genomes and raw sequencing data from the Sequence Read Archive (SRA) for these genera (Fig. S3, Table S7, Methods). By performing de novo assembly and orthologous gene prediction, we identified over 500,000 genes across the dataset, with 534 ortho-groups present in all species. The resulting phylogenomic tree revealed that the Iceman isolates cluster within distinct, well-supported taxonomic lineages, often showing close evolutionary proximity to strains recovered from extreme cold environments such as Antarctica, Arctic glaciers, and high-altitude regions in Italy and Russia (Fig. S3) [9]. This genome-level resolution confirms that these yeasts are not common clinical or food-borne contaminants but rather specialized psychrophilic organisms whose presence on the mummy is likely linked to its unique alpine history and subsequent cold-storage conservation.

### Reconstruction of metagenome-assembled genomes and comparative analysis

To move beyond taxonomic marker genes and resolve the functional and evolutionary characteristics of the Iceman’s microbial community, three representative samples were selected for deep shotgun metagenomic sequencing: internal body water (IW18), finding site soil (Iceman Soil), and an internal skin fragment (ITIIIAB/Skin 2954). The DNA extracted from these samples was converted into double-indexed libraries and subjected to whole-genome shotgun sequencing (Methods).

To provide a broader context and assess microbial stability over time, we integrated this new data with additional publicly available datasets from previous studies on the Iceman [7, 10]. This inclusion of external datasets allowed for a cross-study comparison, ensuring that our reconstructed genomes were representative of the mummy’s long-term microbial profile rather than isolated sampling events (Table S8).

**Fig. 3:**
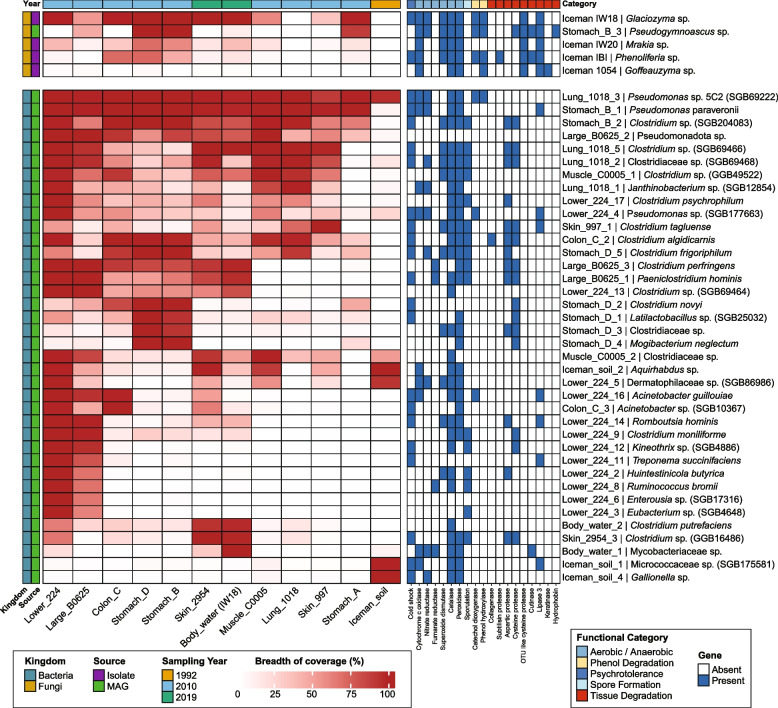
Comparative genomic analysis of microbial MAGs across Iceman samples, showing coverage breadth and functional potential. The heatmap compares the genomic characteristics of 38 bacterial metagenome-assembled genomes (MAGs) and 5 fungal genomes (4 generated from yeast isolated + 1 MAG) recovered from the Iceman and control samples. Left panel: Breadth of coverage (%) for each MAG across different sample types (Iceman tissues, gut content, and environmental controls) and sampling years (1992, 2010, 2019). Color intensity (white to red) indicates increasing coverage breadth, with red representing near-complete genomic coverage. The breadth calculation was done using coverM (Methods). Right panel: Presence or absence of functional gene categories associated with environmental adaptation and potential conservation impact. Functional categories include aerobic/anaerobic metabolism, phenol degradation, psychrotolerance, spore formation, and biomolecule degradation. The labels on the right side refer to MAG IDs and the assigned taxonomy. For further details, please refer to Table S9

Utilizing a multi-tool de novo metagenomic assembly and binning pipeline (Methods), we reconstructed 38 high- and medium-quality bacterial metagenome-assembled genomes (MAGs), with >50% completeness and <10% contamination + one fungal genome with 95% completeness. Next, we screened all samples against all reconstructed genomes (38 bacterial MAGs + 5 fungal genomes: 1 MAG and 4 isolate draft genomes), which revealed a high degree of taxonomic specialization across the sampling sites (Fig. 3, Table S9).

Prominently, *Pseudomonas* spp. were abundant across nearly all samples and years, particularly the *Pseudomonas* sp. 5C2 (SGB69222, taxid 3048588) which showed breadth of coverage > 80% in all samples, including the soil sample from the finding site. Therefore, we performed a high-resolution strain-level analysis to determine if the mummy is colonized by a single persistent strain or multiple environmental introductions (Fig. S5, Methods). Relative abundance and phylogenomic reconstruction based on consensus marker gene sequences revealed that the *Pseudomonas* strains found across different tissues (e.g., lung, stomach, skin) and sampling years are nearly identical to one another (Fig. S5a, b).

Statistical comparison of strain divergence showed that the mutation percentage between strains recovered from different Iceman tissues was significantly lower than the divergence between the Iceman strains and the finding site soil or the MetaPhlAn reference sequence (Wilcoxon rank-sum test) (Fig. S5c). This high degree of genomic similarity indicates that a single, specific strain of *Pseudomonas* sp. 5C2 has successfully colonized the mummy and persists across various anatomical sites. The divergence from the soil-derived *Pseudomonas* further suggests that this specific strain may have adapted to the unique conditions of the conservation facility or the mummy's tissues themselves, rather than being a result of continuous re-introduction from the original finding site environment despite strict decontamination protocols. It is worth mentioning that this species was originally isolated from a cold-adapted environment, i.e., cryoconite in Greenland, which represents similar conditions to the Alpin glaciers [9].

In addition to soil-derived taxa, we identified a persistent “core” community of anaerobic bacteria that dominated internal tissues across all sampling decades. This core mainly consists of diverse members of the Clostridiaceae family, including *Clostridium frigoriphilum*, *C. perfringens*, *C. algidicarnis*, and *C. tagluense*. These species displayed nearly complete genomic coverage in the 1992 and 2010 stomach and lung samples, as well as in the 2019 skin samples, indicating their resilience and suggesting a key role in long-term tissue colonization. Notably, these *Clostridium* species are commonly reported in mummies recovered from cold environments [11].

In contrast, other *Clostridia* (i.e., *C. tagluense*, *C. algidicarnis*, and *C. putrefaciens*) were largely absent from the Iceman’s soil but enriched in internal tissues such as the colon and body water, reflecting both environmental and postmortem sources. Unlike *C. perfringens*, these taxa show no evidence of endogenous origin but are rather commonly found in cold soils, permafrost, and anaerobic micro-environments rich in organic matter, making them well-suited to the glacier environment where the body was preserved. *Clostridium algidicarnis*, known for causing blown-pack spoilage of vacuum-packed chilled meat, may have colonized the mummy’s tissues after death under anaerobic, low-temperature conditions that mimic their typical food-associated habitats [12]. *C. tagluense*, originally described from Arctic permafrost, suggests a likely environmental introduction from the surrounding ice and soil. *C. putrefaciens*, often linked to marine and terrestrial sediments as well as protein-rich food spoilage, could similarly reflect environmental contamination or postmortem proliferation in decaying tissue. Together, these bacteria most likely represent psychrotolerant clostridia from the Iceman’s burial environment rather than his endogenous gut microbiome.

In contrast, the aerobic species *Acinetobacter guillouiae* was mainly present in the intestinal samples (Lower_224, Large_B0625, and Colon_C) as well as the skin sample from 2019. The intestinal samples (Lower_224, Large_B0625, and Colon_C) hosted the most distinct and diverse species, largely absent from any other body site or environmental control. Key species identified here include *Romboutsia hominis*, *Clostridium moniliforme*, *Kineothrix* sp. (SGB4886), and the spirochaete *Treponema succinifaciens*. Additionally, the presence of *Ruminococcus bromii* and *Eubacterium* sp. (SGB4648) further characterizes this community as endogenous remains of the Iceman’s gut microbiome. The high breadth of coverage for these taxa suggests that the intestinal tract remains a largely protected microenvironment, preserving the ancestral microbial signature even after five millennia.

In the 2019 sampling series, the “body water” (IW18) and skin (Skin_2954) showed a significant influx of specialized psychrophilic species. These include the bacterial MAG *Clostridium putrefaciens* and the four isolated yeast species: *Glaciozyma* sp., *Mrakia* sp., *Phenoliferia* sp., and *Goffeauzyma* sp. While *Phenoliferia* and *Goffeauzyma* showed sporadic presence in earlier samples, *Glaciozyma* and *Mrakia* exhibited a dramatic increase in breadth in the most recent 2019 data. This suggests a temporal shift in the microbiome composition.

### Authentication via ancient DNA damage profiling

To distinguish between ancient microbial signatures and modern vegetative growth, we assessed the frequency of cytosine to thymine (C-to-T) deamination at the 5′ termini of the mapped reads (Fig. S5). Significant deamination levels, frequently exceeding 10% and reaching up to 20% in specific samples, were observed for the core anaerobic taxa, including *Clostridium* and *Romboutsia* MAGs. This damage signal was particularly pronounced in the 1992 and 2010 sampling series, confirming that these anaerobic lineages are not modern contaminants but represent an ancient community associated with the Iceman’s long-term taphonomic history. Notably, the gut-exclusive MAGs (e.g., *Treponema succinifaciens* and *Kineothrix* sp.) also exhibited clear deamination profiles, further authenticating their endogenous origin. In contrast, several environmental MAGs and the newly isolated yeasts displayed lower or negligible damage levels in recent samples (e.g., 2019 Body Water), suggesting a more recent proliferation of these taxa within the conservation environment.

### Functional profiles and potential impact on mummy conservation

To assess the potential impact of the mummy-associated microbiota on the conservation, we analyzed the functional gene repertoire across the reconstructed MAGs and yeast genomes (Table S10). The genomic analysis reveals a community that is not merely present but functionally equipped for survival and potential substrate utilization under cold-storage conditions (Fig. 3).

Functional profiling revealed that the recovered microbial community possesses a diverse arsenal of genes geared toward survival in extreme environments and potential interaction with host tissues (Fig. 3). While psychrotolerance is a hallmark of this ecosystem, it is not universally mediated by cold-shock proteins; rather, we observed a mosaic of survival strategies, including the production of cryoprotectants and specialized membrane lipid remodeling. Notably, the presence of genes encoding collagen-degrading enzymes may have implications for the mummy´s structural preservation. For example, *Clostridium algidicarnis* was found to carry a specific collagenase-encoding gene. Given that collagen is a primary structural component of the mummy’s skin and connective tissues, the presence of these genes suggests a indirect risk to the mummy’s integrity. Additionally, a significant proportion of the *Clostridium* MAGs and the yeast isolates (*Glaciozyma* and *Mrakia*) harbor genes associated with other tissue degradation, including proteases and lipases. These enzymatic pathways are critical for breaking down complex organic materials, possibly providing the microbes with the carbon and nitrogen sources necessary for growth within the desiccated, mummified tissues.

Crucially, our analysis revealed that several key taxa possess the functional pathways for phenol degradation, specifically the genes for phenol hydroxylase and catechol dioxygenase. This catabolic potential was identified in *Pseudomonas* sp. 5C2, as well as the psychrotolerant fungal isolates *Glaciozyma watsonii*, *Pseudogymnoascus pannorum*, and *Phenoliferia glacialis*. The ability to degrade phenols, which were used in the Iceman’s historical conservation and persist as degradation byproducts [1, 2], underscores the metabolic versatility of these microbes and possibly displays a selective advantage for the upgrowth of phenol-degrading microorganisms. Coupled with the capacity for endospore formation in *Clostridiaceae*—a dormant state that protects the microbial genome against desiccation and chemical stress—these findings indicate that the microbiome possesses the metabolic toolkit required to actively interact with the host and withstand disinfection protocols.

### Temporal dynamics and authentication of the Iceman yeast microbiota

**Fig. 4:**
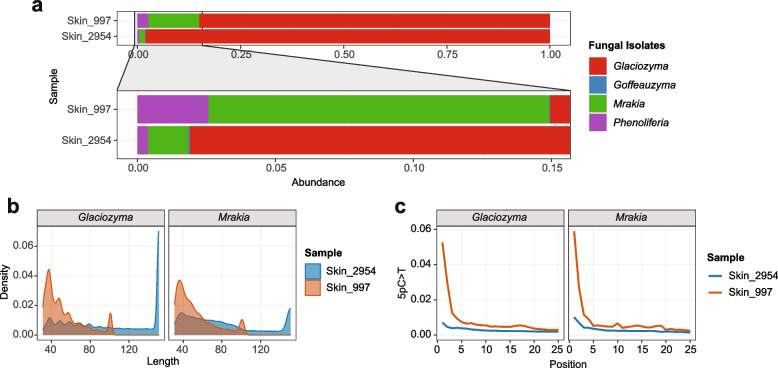
Changes in yeast abundances in the Iceman skin tissues over time. **a** Relative abundances of the Iceman yeast isolated *Glaciozyma* (IW18), *Goffeauzyma* (1054), *Mrakia* (IW20), and *Phenoliferia* (IBI) in the skin tissue samples (Skin_997, sampled in 2010 and Skin_2954, sampled in 2019), showing overall yeast composition, with zoom-in on the low abundant portion. **b** Density plot showing the fragment length distributions of reads mapped to each isolate in Skin_997 and Skin_2954. **c** DNA damage profiles in reads assigned to each isolate genome (5′-end cytosine-to-thymine (5pC > T) substitution frequency). Notice the difference in length distribution and damage levels between 2010 and 2019. The analysis was based on competitive mapping of short reads against the yeast draft genomes using Bowtie2, followed by mapping quality filtering (MAPQ ≥ 20) using SAMtools and ancient DNA damage estimation via DamageProfiler. To determine species composition, raw fungal read counts were first normalized by the total number of paired-end reads per sample to account for varying sequencing depths. These normalized values were then divided by the sum of all normalized fungal abundances within each sample to calculate the final relative abundance per species

To assess whether yeast communities on the Iceman’s skin have changed over time under the current conservation conditions, we compared two skin samples collected at different time points: Skin_997 (sampled in 2010) and Skin_2954 (sampled in 2019). The abundance analysis revealed a striking temporal shift in yeast composition (Fig. 4). While both samples harbor detectable levels of *Goffeauzyma*, *Mrakia*, and *Phenoliferia*, *Glaciozyma* (represented by isolate IW18) showed a marked increase in relative abundance in the 2019 sample, rising from 85% in 2010 to 98%, becoming the dominant yeast strain in 2019, suggesting not only increased detection but also greater genomic representation—indicative of continuous proliferation.

To evaluate whether this shift reflects biological activity or post-mortem degradation patterns, we checked fragment length distributions and DNA damage profiles for reads mapped to each isolate in both skin samples (Fig. 4b, c). In Skin_997 (2010), the peak of fragment lengths for all four yeasts were short (~ 40 nt), with C-to-T substitution frequencies of ~ 5%. In contrast, Skin_2954 (2019) exhibited longer average fragment lengths and reduced damage signatures, particularly for *Glaciozyma*. This pattern is inconsistent with passive accumulation of degraded DNA over time and instead suggests recent or ongoing microbial activity, potentially driven by localized microhabitat changes on the skin surface.

Together, these findings indicate that the yeast community on the Iceman’s skin is not static. The substantial rise of *Glaciozyma* between 2010 and 2019, coupled with its reduced DNA damage and longer fragments (Fig. 4), strongly implies that this cold-adapted yeast may be metabolically active or at least capable of replication under current conservation conditions (−6 °C).

Furthermore, the application of Post-Mortem Damage (PMD) filtering confirmed this temporal transition. As we increased the PMD score threshold, the percentage of retained reads for all four yeast genera decreased significantly across all samples, but the rate of decrease varied according to the biomass of viable cells present (Fig. S6). Samples with a higher proportion of living, modern cells showed a sharper drop-off in read retention after filtering, as modern DNA lacks the characteristic damage profile of ancient samples.

## Discussion

Our comprehensive assessment reveals that the Iceman is not a biologically “frozen” time-capsule but rather a complex ecosystem shaped by three distinct temporal forces: original alpine glacier infiltration, endogenous post-mortem succession, and three decades of modern conservation. We identified four primary entry routes: (i) museum infrastructure, (ii) human-associated introduction, (iii) spray water, and (iv) glacier-derived persistence—that together challenge the assumption that −6 °C storage fully halts microbial life. The current conservation regime acts as a powerful selective filter; for instance, routine UV-treated spray water introduced a dominant *Methylobacterium* and *Sphingomonas* signature post-2010. These taxa, known for biofilm formation and environmental resilience [13–16], have effectively reshaped the mummy’s external microbiome, illustrating how even humidification treatment can drive anthropogenic microbial turnover.

The interplay between chemical stressors and microbial adaptation is most evident in the legacy of phenol treatments. The historical use of phenol as a disinfectant created a selective micro-environment that favored specialized degraders. Our results confirm that *Pseudomonas* sp. 5C2 and psychrophilic fungal isolates like *Phenoliferia glacialis* (originally an alpine sediment dweller) possess pathways necessary to thrive in this micro-environment. Similarly, the presence of *Acinetobacter* and *Staphylococcus* on the external surface—taxa capable of tolerating common disinfectants [17–19]—suggests these microbes survived museum sterilization campaigns by exploiting small, protected niches on the body surface. This highlights an unintended selective pressure where standard preservation interventions, such as chemical disinfection and humidification, facilitate the persistence of metabolically versatile taxa that are uniquely adapted to exploit micro-environments on the mummy’s surface.

Most interestingly, we isolated four cold-adapted yeasts, belonging to the genera *Phenoliferia*, *Glaciozyma*, *Goffeauzyma*, and *Mrakia*, directly from the mummy’s skin, stomach, and body water. Their detection in shotgun metagenomes, coupled with ancient DNA damage profiles, strongly supports their authentic, ancient origin, either as dormant survivors or descendants of post-mortem colonizers. Although reviving ancient microbes after millennia has been a topic of debate for long times [20–22], there exist some recent reports that demonstrated microbial survival for similar or even much longer times [23, 24]. These species are exclusively reported from glacial and alpine environments, including cryoconite holes near the Iceman’s discovery site [25, 26], indicating potential postmortem infiltration through the mummy’s natural openings. The critical question that imposes itself now is whether these yeasts are descendants of ancient yeasts that maintained their multiplication along the years, or they were in a dormant state that was revived after thawing the mummy.

Our findings resolve the paradox of “culturable ancient DNA.” By comparing 2010 and 2019 samples, we observed that the psychrophilic yeast *Glaciozyma* transitioned to dominance alongside lengthening DNA fragments and reduced damage profiles. This suggests that glacier-derived microbes, such as *Glaciozyma* and *Pseudomonas* sp. 5C2, are not merely dormant relics but may be engaged in slow, persistent proliferation within micro-environments of transient moisture. This active metabolism may pose a direct threat to the mummy’s structural integrity, particularly given the identification of lipase and proteinase-encoding genes.

Despite the comprehensive multi-omics framework employed in this study, several limitations must be acknowledged alongside avenues for future investigation. The irreplaceable and ethically constrained nature of the Iceman mummy prevents replicate sampling from individual anatomical sites, limiting statistical power for within-site comparisons. Future non-invasive sampling strategies, e.g., systematic collection of thawed water during routine defrosting events, may offer a practical method to increase sampling depth without further disturbing the mummy’s tissues. While MAG-based functional profiling provides genomic evidence for degradative potential, it cannot distinguish between metabolically active and dormant cells. The integration of meta-transcriptomics (RNA-seq) would be a critical next step, enabling direct measurement of active gene expression under current conservation conditions and transforming the present risk assessment from a genomic potential framework into one grounded in demonstrated in situ activity. Complementarily, the application of propidium monoazide (PMA) treatment prior to DNA extraction would provide a more stringent viability filter, helping to disentangle living from dead microbial fractions, particularly in the low-biomass swab and water samples analyzed here. The identification of phenol-degradation genes in *Pseudomonas* sp. 5C2 and several psychrophilic yeast isolates further raises the possibility that historical phenol-based conservation treatments may have exerted a selective pressure shaping the current microbial community, a hypothesis that could be tested through targeted enrichment cultures or competitive fitness assays using the already-isolated strains. Finally, the 2010–2019 temporal comparison of skin-associated yeast communities, while informative, is restricted to two timepoints; establishing a longitudinal monitoring program incorporating periodic low-invasive sampling combined with both amplicon and shotgun sequencing would enable systematic tracking of community dynamics and early detection of conservation-relevant microbial shifts. More broadly, our study highlights the limitations of using DNA damage profiles in isolation to determine microbial “authenticity.” The coexistence of damaged, ancient DNA with viable, culturable cells of the same lineage suggests that selective pressures and microhabitat variability can result in heterogeneous damage patterns, whereby relying solely on deamination signatures risks misclassifying glacier-derived persistence as modern contamination, or vice versa. We therefore advocate for a multi-source framework—integrating strain-level analysis, environmental controls such as burial soil, and functional genomic potential—as the most robust approach to accurately disentangling the Iceman's complex taphonomic history from the ongoing biological impacts on its conservation.

## Conclusions

The Iceman mummy is not a static artifact but a dynamic ecosystem of living archive where ancient glacier-derived microbes and modern contaminants coexist under museum conditions. Our integrated microbiome analysis framework reveals that current conservation parameters (−6 °C, 99% RH) suppress most decomposers yet sustain psychrophilic taxa with genomic signatures of viability and latent degradative potential. Critically, we correct prior misclassifications: soil-derived *Pseudomonas* and cold-adapted fungi are authentic taphonomic components, not modern contaminants, while human-introduced taxa (*Methylobacterium*, *Sphingomonas*) now dominate external surfaces. These findings necessitate a paradigm shift: conservation must move beyond static preservation to proactive genomic surveillance. By tracking indicator taxa, integrating environmental baselines, and adopting periodic multi-omics monitoring, we can detect transitions from dormancy to activity before shifts in informations occurs, ensuring this irreplaceable window into the Copper Age endures for future generations.

## Methods

### Sample collection

The sampling campaign started on 11th of March 2019, after taking all necessary permissions from the South Tyrol Archeological Museum in Bolzano, Italy. On the 29th of April 2019, the Iceman mummy was defrosted by taking it out of the conservation chamber to the neighboring lab (Figure S1). The mummy was kept at 4 °C and for 5 h until the outer ice layer, covering the whole body, fully defrosted. The surface ice pieces/blocks as well as the thawed water were gradually collected over the time of defrosting (Figure S2). Then, the whole-body surface was swabbed into different samples, using eSwab sampling devices (COPAN©). Moreover, a few soft tissue samples were taken from the exposed internal parts of the body (Figure S2, Table S1). Additionally, the previous stomach content sample (Eurac ID 1054) hosted at the Institute for Mummy Studies of Eurac Research and sampled in 2010 was included in this study for culture-dependent analysis [7, 10]. Additionally, soil samples that were collected from underneath the mummy during the excavation campaign in 1991 were also included (Table S1).

### Culture-independent analysis

#### DNA extraction

DNA was extracted from swab, tissue, and soil samples using the CTAB/LPA protocol [27]. While water samples were initially filtered through 0.45 µm Sterivex™ filter units to ensure capturing cellular DNA, the DNeasy Power Water Kit was used for further DNA extraction steps. DNA concentrations were measured using the Quantus Fluorometer (Promega, WI, USA).

#### Amplicon-based microbiome profiling

Initially, we screened all samples for the presence of full-length 16S rRNA, using 35 cycles of PCR amplification. Second, samples that showed a positive PCR band on agarose gel were selected for another PCR amplification for the V3/V4 regions of the 16S rRNA genes (30 cycles). The primers and thermal cycling programs are described in Table S2.

For the water samples (SWColl, IIce, and IWColl), we could not retrieve any full-length 16S rRNA gene amplicons from the DNA extracts; however, as soon as we increased the filtered volume from 10 to 200 ml, we got positive bands, with even fewer number of amplification cycles. This implies that the analysis is influenced by the initial biomass and hence the filtered water volume.

The PCR products were then purified with MiniElute PCR purification kit (Qiagen, Germany). Third, the purified PCR products (16S rRNA) were converted into libraries according to the protocol described for ancient DNA [28]. Then, the produced libraries were double indexed and quantified using Quantus Fluorometer (Promega, WI, USA). Finally, libraries were pooled in equimolar amounts and sequenced using Illumina MiSeq (300 × 2) (Table S3).

To rigorously account for the low-biomass nature of our samples, we included 6 extraction blank controls, representing the swabs, water filtration tools, and DNA extraction reagents, processed in parallel across all experimental batches. Every blank control was carried through the complete library preparation and sequencing pipeline, irrespective of PCR yield or quantification results. These sequences were subsequently utilized to establish a contamination threshold, allowing for the precise removal of exogenous signals and ensuring the authenticity of the microbial profiles recovered from the mummy’s tissues and environmental sources.

After sequencing, forward and reverse reads were merged using the “*fastq_mergepairs*” command of usearch [29], then the command “*fastq_filter*” of vsearch tool [30], where 261,159 sequences were discarded, and 812,283 sequences passed the quality filter. We further used the command “*derep_fulllength*” of the vsearch tool to dereplicate the sequences which resulted in 187,625 unique sequences. Finally, we used the unoise3 tool and “*usearch_global*” command of vsearch tool to generate amplicon sequence variants (ASV) table, with the option of minimum support percentage of 0.01% of the total number of unique sequences and sequence identity of 99%. The taxonomic assignment of the ASVs was carried out using sintax classifier within usearch with a cutoff of 0.9 and using the ribosomal database project (RDP database) 16S_v16 training set for 16S rRNA genes [31, 32] (Table S4). We used MAFFT tool to perform the multiple sequence alignment (MSA) and phylogenetic analysis of the ASVs [33].

The produced ASV table, taxonomy tables, phylogeny file, as well as the related metadata were combined to produce the phyloseq object in R, which was used for the microbiome analysis, using the R-package “phyloseq” [34]. All ASVs detected in any negative control samples (extraction blanks or library preparation blanks) were excluded from downstream analysis to eliminate potential contaminants. The package “*ggplot2*” was used to generate the plots in R (v. 4.2.0). The Linear discriminant analysis Effect Size (LEfSe) was used to infer the significantly different taxa among the tested sample groups [35].

### Culture-dependent analysis

The culture-dependent analysis targeted two types of samples. First, the aerial spores from the museum environments and the internal air of the Iceman conservation chamber. Second, the samples that were collected directly from the mummy or in contact with the mummy. So, initially, we collected air-borne microbial spores and cells from the Iceman chamber and laboratory (Figure S1), as well as the control room nearby the Iceman chamber, using SAS microbial air sampler (Cherwell laboratories, Bicester, UK). The volumes of the collected air were equivalent to the total volume of air present in the sampled room. The spores were directly collected on agar culture media of Malt-extract agar (MEA) and Dichloran-Glycerol (DG18) and incubated at 22 °C, and microbial growth was monitored periodically over 14 days (Table S5).

Swab samples were directly streaked on agar plates of Brain-Heart Infusion (BHI), Reasoner's 2A (R2A), and Malt-Extract Agar (MEA). Plates were incubated at two different incubation temperatures of 4 °C and 20–22 °C, for 28 days. For the checked internal water samples, 100 µl were spread on agar plates of the same media. For the stomach sample 1054, 0.1 g of the content were enriched in Brain-Heart Infusion (BHI) broth and after 2 weeks of incubation at 4 °C, aliquots of 100 µl were spread on agar culture media of BHI for single colony isolation. The emerging colonies were then sub-cultured and checked microscopically.

Then, fungal mycelia and yeast and bacterial colonies were harvested from pure culture agar plates and used for genomic DNA extraction using QIAamp Fast DNA Stool Mini Kit (Qiagen, Germany). For microbial identification, the internal transcribed spacers (ITS) and full-length 16S rRNA genes were amplified from fungal and bacterial DNA extracts, respectively, using AccuPrime Pfx SuperMix (Thermo Fisher Scientific, USA), and universal bacterial and fungal primers (Table S2). The amplified products were checked for the right fragment sizes on 1.5% agarose gel, and purified using MiniElute PCR purification kit (Qiagen, Germany). Finally, the purified products were sequenced using the automated Sanger sequencing technology service of Eurofins Genomics Inc. (Mix2Seq, eurofinsgenomics.eu).

For taxonomic assignment of the sequenced isolates, we performed nucleotide BLAST searches (BLASTn; https://blast.ncbi.nlm.nih.gov/) against the NCBI non-redundant nucleotide (nt) database [36] (Table S3). For each query sequence, we retrieved the top-scoring hits along with representative reference sequences from closely related species to enable robust phylogenetic placement. These sequences were aligned using MAFFT with default parameters to generate high-quality multiple sequence alignments. To improve alignment accuracy and reduce noise, poorly aligned regions and positions containing excessive gaps were trimmed using TrimAl v1.4 (https://github.com/inab/trimal) under the “automated1” heuristic setting. Phylogenetic trees were then inferred using IQ-TREE v3 [37] with automatic model selection (ModelFinder) and branch support assessed via ultrafast bootstrap (UFBoot, 1000 replicates).

### Yeast genome analysis

The extracted DNA from the yeast isolates was converted into double-indexed libraries [28]. The libraries were pooled and subjected to whole-genome shotgun sequencing in the Illumina HiSeqX platform 150 × 2 paired-end (PE). The generated paired-end files for isolates cultivated in this study or other sequencing data obtained from the short reads archive (SRA) database were quality-checked using fastp [38] to trim the adapters and to discard the low-quality sequences and sites. Then, de novo genome assembly was performed using SPAdes assembler using the “–isolate” mode [39]. An additional binning step using MetaBAT2 [40] was performed for the resulting contigs for each isolate to exclude potential contaminants and contigs below 1500 nt. The completeness of the resulting genomes was assessed using BUSCO [41] and FGMP [42] (Table S6).

### Shotgun metagenomic analysis

Three samples were further analyzed with shotgun metagenomic sequencing: IW18, Soil, and ITIIIAB (Skin 2954). The extracted DNA from those samples was converted into double indexed libraries [28]. The libraries were pooled and subjected to whole-genome shotgun sequencing in Illumina HiSeqX platform 150 × 2 paired-end (PE) (Table S8).

Additional publicly available datasets from previous studies on the Iceman were included in the analysis for temporal comparison [7, 10]. Initially, raw reads were processed through a quality-control pipeline to remove adapters and low-quality bases using fastp. The metagenomic assembly was performed using MEGAHIT [43] and metaSPAdes [39] to generate high-quality contigs. Following assembly, we employed a multi-tool binning strategy to maximize the recovery of metagenome-assembled genomes (MAGs). Initial binning was conducted using MetaBAT2 [40], MaxBin2 [44], and CONCOCT [45]. To generate a consensus set of high-quality MAGs, we integrated these individual outputs using DAS Tool [46]. Then, all resulted bins (from DAS Tool or from the used binners) were checked for completeness and contamination using CheckM2 [47] then bins with completeness > 50% and contamination < 10% were dereplicated at an average nucleotide identity of 95% using dRep. Overall, 38 bacterial bins were kept for further analysis (Table S9). In parallel, the bins with genome size of > 15 Mbps were suspected to be of eukaryotic origin and checked for completeness using BUSCO [41] with “–*auto*-*lineage*-*euk*”. Then, taxonomic assignment was carried out using kraken2 against the NCBI-nt database [48].

Taxonomic classification for the bacterial bins was performed using GTDBtk [49] as well as PhyloPhlAn 3 [50] using “*phylophlan_assign_sgbs*”.

To determine the relative abundance and breadth of coverage of the identified taxa across different samples, we utilized CoverM [51]. Read alignments were filtered to retain only high-confidence mappings with a minimum mapping quality (MAPQ) score of 20. This threshold, calculated as (where is the probability of a mismatched alignment), ensures alignment accuracy and excludes reads with significant multi-mapping ambiguity [52].

The resulting BAM files were used to assess the ancient DNA damage (i.e., deamination frequency of terminal cytosine to thymine) using DamageProfiler [53].

### Functional annotation of microbial genomes

Prokka (v1.14.6) was used for gene prediction on bacterial metagenome-assembled genomes (MAGs; *n* = 38) with default parameters [54], while funannotate (v1.8.1) was used for fungal genomes (*n* = 5: 4 isolate draft genomes + 1 MAG) after masking repetitive regions [55]. Then eggNOG-mapper (v2.1.12) [56] was used to annotate all resulting protein sequences against the eggNOG database [57], assigning orthologous groups, Gene Ontology terms, KEGG pathways, and KEGG modules. Then, we systematically screened outputs for genes associated with the following: (i) phenol degradation (phenol hydroxylase, catechol dioxygenase); (ii) tissue degradation potential (collagenase, subtilisin protease, subtilisin-like protease, aspartic protease, cysteine protease, OTU-like cysteine protease, cutinase, Lipase_3); (iii) keratin degradation (sulphite efflux pump in fungi, hydrophobins); (iv) cold adaptation (cold-shock proteins, trehalose synthase); (v) sporulation (bacterial *spo0A*); and (vi) metabolic lifestyle (cytochrome c oxidase, nitrate reductase, fumarate reductase, superoxide dismutase, catalase, peroxidase). Gene presence was validated by manual conserved domain checks (Pfam), KEGG ortholog assignments, and literature-supported functional criteria (Table S10).

### Taxonomic profiling and strain-level resolution

To achieve high-resolution genomic tracking, we performed strain-level analysis using the StrainPhlAn module within the MetaPhlAn4 (v4.2.2) [38]. Paired-end reads were mapped against the CHOCOPhlAn database (vJan25) to generate species-level genome bin assignments. For taxa meeting a minimum coverage threshold of 3×, StrainPhlAn reconstructed marker-based strain haplotypes from the generated SAM files. This approach allowed us to resolve the fine-scale genetic architecture of persistent colonizers, such as *Pseudomonas* sp. 5C2, by comparing reconstructed haplotypes across anatomical sites and environmental controls. The resulting phylogenetic trees, visualized through iTOL.

Statistical analyses and visualizations were conducted in R (v4.2.0) using the tidyverse, phyloseq, and ggplot2 packages.

## Supplementary Information

Additional file 1. Supplementary tables.

Additional file 2. Supplementary figures. Figure S1. Iceman chamber spore sampling. Schematic floor plan of the Iceman conservation facility at the South Tyrolean Museum of Archaeology in Bolzano, Italy. The main chamber (where the mummy is stored) is connected to an antechamber, a chamber laboratory, a backup chamber, and a control and monitoring room. Air spore sampling (indicated by wind icons) was conducted in multiple zones to assess microbial load across the environment. Figure S2. Sampling procedures for microbiological analysis of the Iceman. (a) Collection of air-borne spores in the conservation chamber. (b) Collection of mummy water from body cavities using a sterile pipette, followed by transfer into a sterile container for storage and downstream analysis. (c) Dissection of soft tissue fragments (e.g., muscle or connective tissue) using sterile surgical tools under controlled conditions. (d) Swabbing of skin surfaces with a sterile cotton-tipped swab to collect microbial material from the outer tissues. All sampling was performed under strict aseptic protocols to ensure the integrity of microbiological data. Figure S3. Phylogenomic tree of the Iceman yeast isolates within reference genomes and publicly deposited related SRA records. The analysis included the 4 draft genomes generated for the 4 isolates from the Iceman mummy as well as the available NCBI reference genomes within the genera: *Mrakia*, *Phenoliferia*, *Glaciozyma*, and *Goffeauzyma*. Due to the limited number of available genomes, we also retrieved raw sequencing data available in the SRA for the same genera and performed de novo assembly following the same procedures we used for the Iceman yeast draft genomes (Methods). To perform phylogenetic analysis of the yeast at genome-level, the tool funannotate (https://github.com/nextgenusfs/funannotate) was used to perform gene prediction and annotations. Then, the amino acid sequences for each isolate were compared against each other to find the orthologous genes using OrthoFinder [55]. OrthoFinder assigned 522,379 genes (98.1% of total) to 19,474 ortho-groups. Fifty percent of all genes were in ortho-groups with 69 or more genes (G50 was 69) and were contained in the largest 2731 ortho-groups (O50 was 2731). There were 534 ortho-groups with all species present and 6 of these consisted entirely of single-copy genes. OrthoFinder then uses dendroblast [59] to infer the phylogenetic distances between isolates. The resulting phylogenies were visualized and annotated using iTOL [60]. Iceman isolates (in red) cluster within distinct taxonomic lineages. Outer colored bars indicate geographic origin of reference strains, while inner colored circles denote taxonomic family and order. Data sources include both GenBank and SRA. Figure S4. Comparison of *Pseudomonas* sp. 5C2 across different samples. (a) Relative abundance of *Pseudomonas* sp. 5C2 (SGB69222) across samples. (b) Strain-level comparison between *Pseudomonas* sp. 5C2. In different samples. Phylogenies are constructed from the consensus sequences of MetaPhlAn markers genes, using StrainPhlAn v4 (Methods). (c) Statistical comparison of strain divergence. Samples are grouped as follows: Reference (The reference sequence of MetaPhlAn v4 “t__SGB69222”), and all Iceman derived samples (tissues). The significance of the differences between the comparison groups was determined using Wilcoxon rank-sum test, and the p-values was adjusted using Bonferroni method Figure S5. Ancient DNA damage of the analyzed genomes. The heatmap shows the deamination frequency of cytosine to thymine at the first position of the mapped reads. The short reads of each sample were mapped against all genomes and metagenome-assembled genomes (MAGs) and the deamination was calculated for each sample and each genome independently. The genomes with < 5% breadth of coverage were excluded from this analysis and are shown in gray color. Figure S6: Impact of PMD filtering on yeast read retention across Iceman samples. The plots show the relative percentage of reads retained for Iceman yeasts (*Glaciozyma*, *Goffeauzyma*, *Mrakia*, *Phenoliferia*) as a function of increasing PMD (Post-Mortem Damage) score threshold, ranging from “null” (no filtering) to 5. Each row corresponds to a distinct Iceman sample (e.g., body water, colon, skin, stomach), with the y-axis displayed on a logarithmic scale to emphasize low-abundance changes. The samples display different patterns of decrease in reads retained after filtering, as this correlates with the biomass of living cells. The analysis was done by mapping the short reads of all samples against the yeast draft genomes using bowtie2 [61] and filtered for minimum mapping quality of 20 using SAMtools [52]. Then PMDtool was applied with different scores ranging from -2 to 5 [62] and ancient DNA reads proportions were counted.

## Data Availability

The sequencing data generated in this study is publicly available at ENA under BioProject: PRJEB94382, adhering to the data reuse guidelines presented in Hug et al. 2025 [58].
