# Supplementary material for: The Iceman’s microbiome: unveiling millennia of microbial diversity and continuity
Source: Microbiome. 2026 Jun 3;14:135. doi: 10.1186/s40168-026-02417-6 (PMC13231656; doi:10.1186/s40168-026-02417-6)
Supplement: Supplementary file 3 — Additional file 2. Supplementary figures. Figure S1. Iceman chamber spore sampling. Schematic floor plan of the Iceman conservation facility at the South Tyrolean Museum of Archaeology in Bolzano, Italy. The main chamber (where the mummy is stored) is connected to an antechamber, a chamber laboratory, a backup chamber, and a control and monitoring room. Air spore sampling (indicated by wind icons) was conducted in multiple zones to assess microbial load across the environment. Figure S2. Sampling procedures for microbiological analysis of the Iceman. (a) Collection of air-borne spores in the conservation chamber. (b) Collection of mummy water from body cavities using a sterile pipette, followed by transfer into a sterile container for storage and downstream analysis. (c) Dissection of soft tissue fragments (e.g., muscle or connective tissue) using sterile surgical tools under controlled conditions. (d) Swabbing of skin surfaces with a sterile cotton-tipped swab to collect microbial material from the outer tissues. All sampling was performed under strict aseptic protocols to ensure the integrity of microbiological data. Figure S3. Phylogenomic tree of the Iceman yeast isolates within reference genomes and publicly deposited related SRA records. The analysis included the 4 draft genomes generated for the 4 isolates from the Iceman mummy as well as the available NCBI reference genomes within the genera: Mrakia, Phenoliferia, Glaciozyma, and Goffeauzyma. Due to the limited number of available genomes, we also retrieved raw sequencing data available in the SRA for the same genera and performed de novo assembly following the same procedures we used for the Iceman yeast draft genomes (Methods). To perform phylogenetic analysis of the yeast at genome-level, the tool funannotate (https://github.com/nextgenusfs/funannotate) was used to perform gene prediction and annotations. Then, the amino acid sequences for each isolate were compared against each other to find the ortholog [file 40168_2026_2417_MOESM2_ESM.docx]

**Supplementary figures**


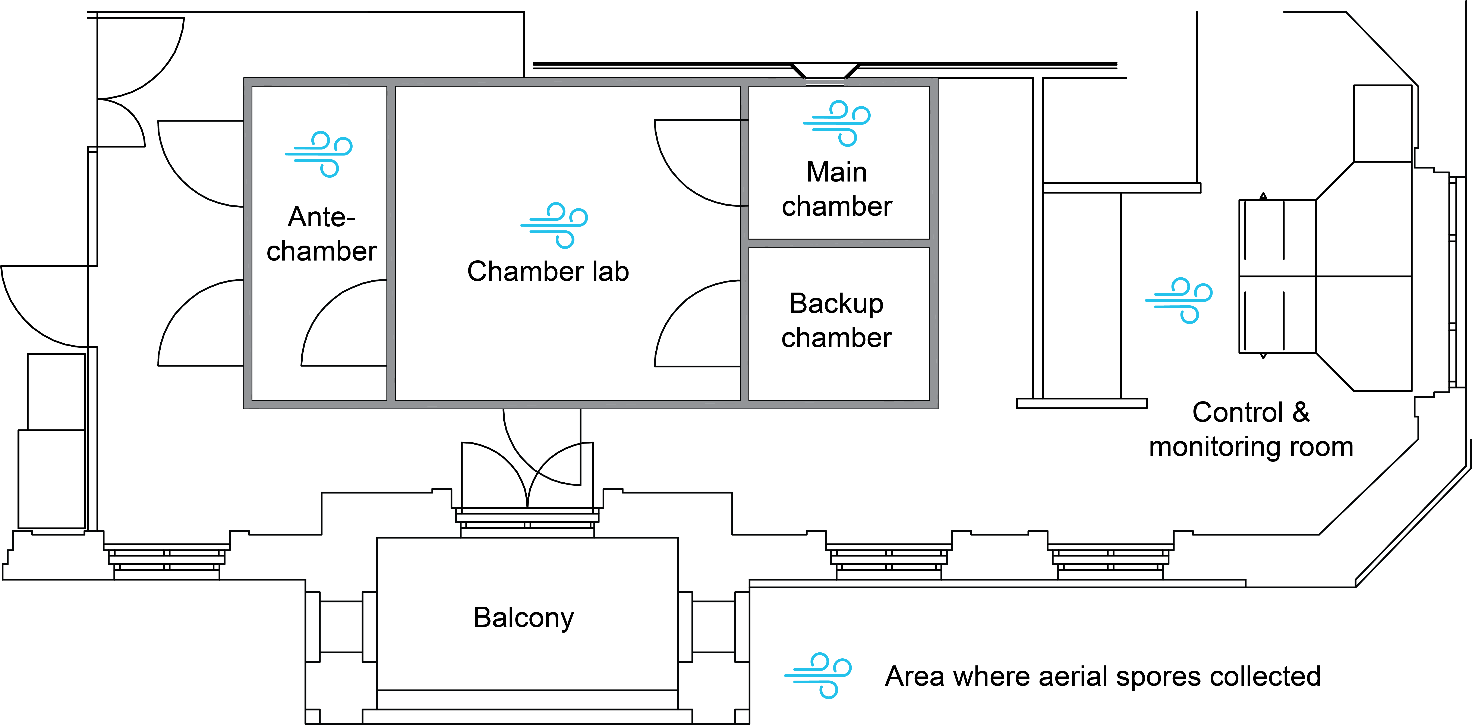


**Figure S1. Iceman chamber spore sampling.** Schematic floor plan of the Iceman conservation facility at the South Tyrolean Museum of Archaeology in Bolzano, Italy. The main chamber (where the mummy is stored) is connected to an antechamber, a chamber laboratory, a backup chamber, and a control and monitoring room. Air spore sampling (indicated by wind icons) was conducted in multiple zones to assess microbial load across the environment.

**
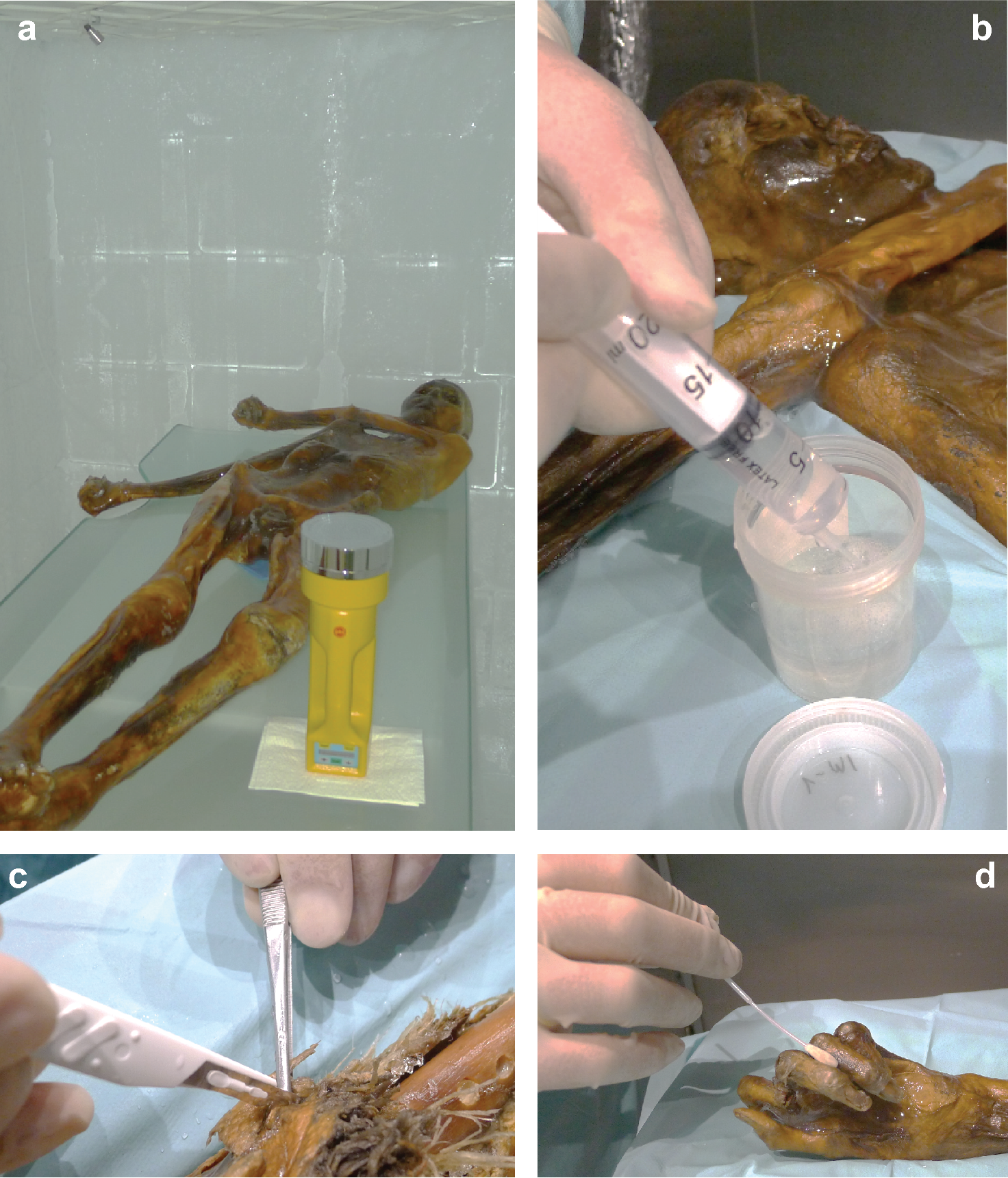
**

**Figure S2. Sampling procedures for microbiological analysis of the Iceman.** (**a**) Collection of air-borne spores in the conservation chamber. (**b**) Collection of mummy water from body cavities using a sterile pipette, followed by transfer into a sterile container for storage and downstream analysis. (**c**) Dissection of soft tissue fragments (e.g., muscle or connective tissue) using sterile surgical tools under controlled conditions. (**d**) Swabbing of skin surfaces with a sterile cotton-tipped swab to collect microbial material from the outer tissues. All sampling was performed under strict aseptic protocols to ensure the integrity of microbiological data.

**
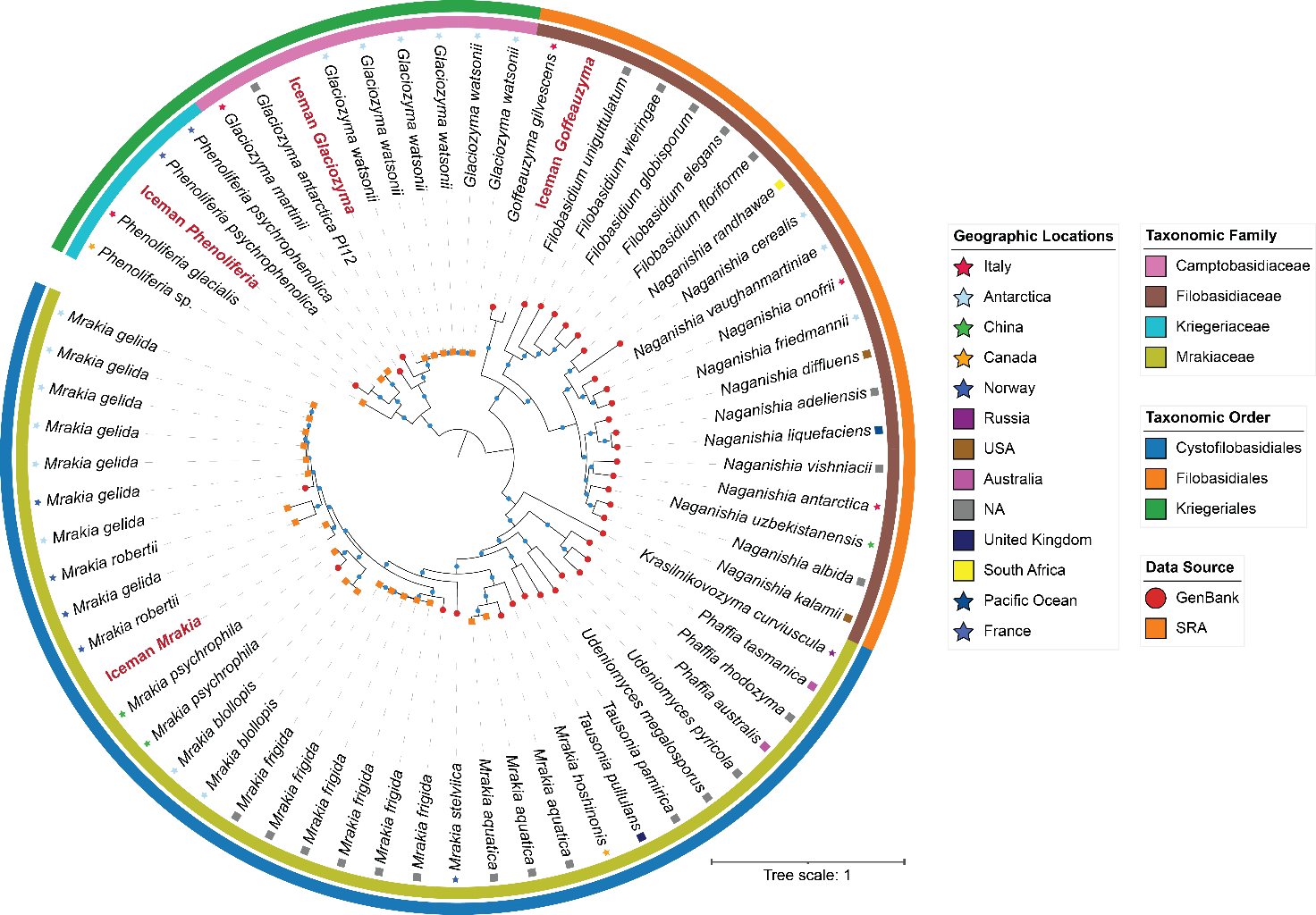
**

**Figure S3. Phylogenomic tree of the Iceman yeast isolates within reference genomes and publicly deposited related SRA records.** The analysis included the 4 draft genomes generated for the 4 isolates from the Iceman mummy as well as the available NCBI reference genomes within the genera: *Mrakia*, *Phenoliferia*, *Glaciozyma*, and *Goffeauzyma*. Due to the limited number of available genomes, we also retrieved raw sequencing data available in the SRA for the same genera and performed de novo assembly following the same procedures we used for the Iceman yeast draft genomes (Methods). To perform phylogenetic analysis of the yeast at genome-level, the tool funannotate (<https://github.com/nextgenusfs/funannotate>) was used to perform gene prediction and annotations. Then, the amino acid sequences for each isolate were compared against each other to find the orthologous genes using OrthoFinder [55]. OrthoFinder assigned 522379 genes (98.1% of total) to 19474 ortho-groups. Fifty percent of all genes were in ortho-groups with 69 or more genes (G50 was 69) and were contained in the largest 2731 ortho-groups (O50 was 2731). There were 534 ortho-groups with all species present and 6 of these consisted entirely of single-copy genes. OrthoFinder then uses dendroblast [59] to infer the phylogenetic distances between isolates. The resulting phylogenies were visualized and annotated using iTOL [60]. Iceman isolates (in red) cluster within distinct taxonomic lineages. Outer colored bars indicate geographic origin of reference strains, while inner colored circles denote taxonomic family and order. Data sources include both GenBank and SRA.

**
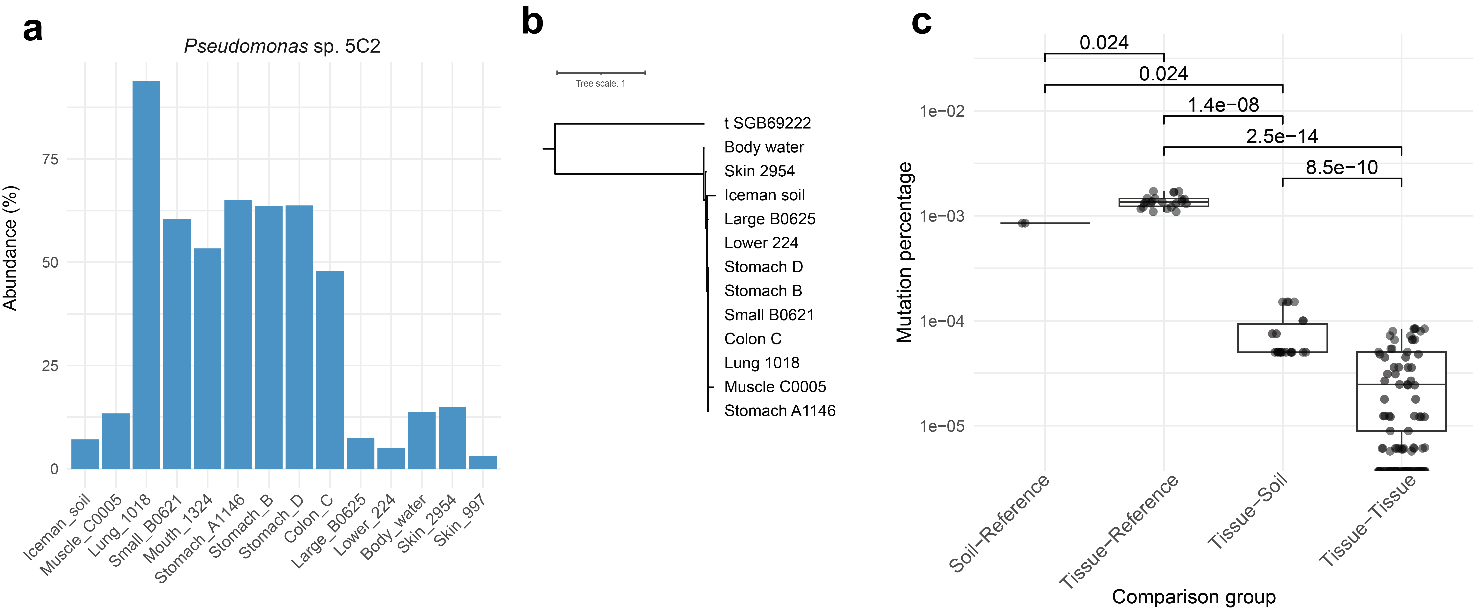
 Figure S4. Comparison of *Pseudomonas* sp. 5C2 across different samples.** (**a**) Relative abundance of *Pseudomonas* sp. 5C2 (SGB69222) across samples. (**b**) Strain-level comparison between *Pseudomonas* sp. 5C2. In different samples. Phylogenies are constructed from the consensus sequences of MetaPhlAn markers genes, using StrainPhlAn v4 (**Methods**). (**c**) Statistical comparison of strain divergence. Samples are grouped as follows: Reference (The reference sequence of MetaPhlAn v4 “t__SGB69222”), and all Iceman derived samples (tissues). The significance of the differences between the comparison groups was determined using Wilcoxon rank-sum test, and the p-values was adjusted using Bonferroni method.

**
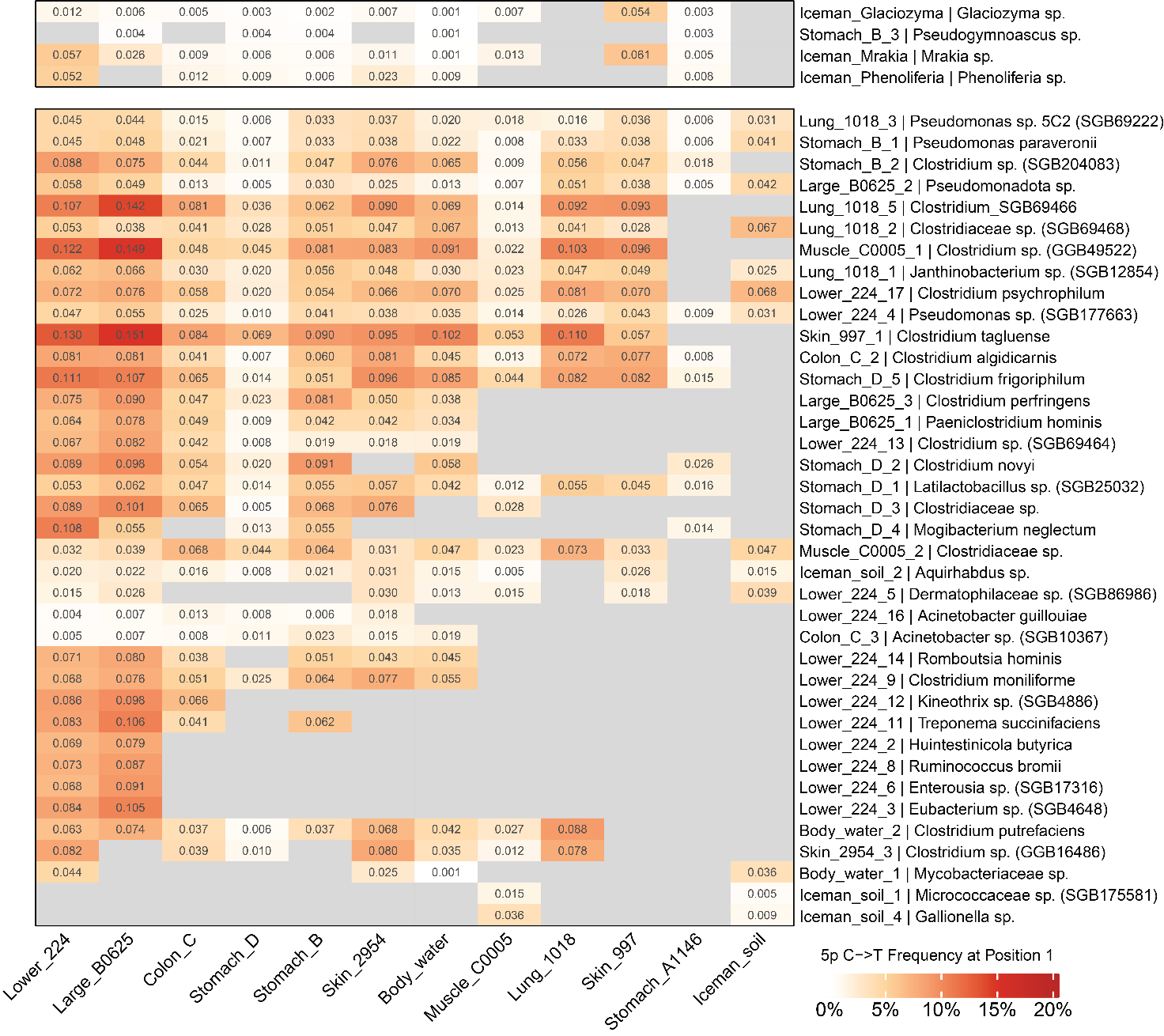
Figure S5. Ancient DNA damage of the analyzed genomes.** The heatmap shows the deamination frequency of cytosine to thymine at the first position of the mapped reads. The short reads of each sample were mapped against all genomes and metagenome-assembled genomes (MAGs) and the de-amination was calculated for each sample and each genome independently. The genomes with < 5% breadth of coverage were excluded from this analysis and are shown in grey color.

**
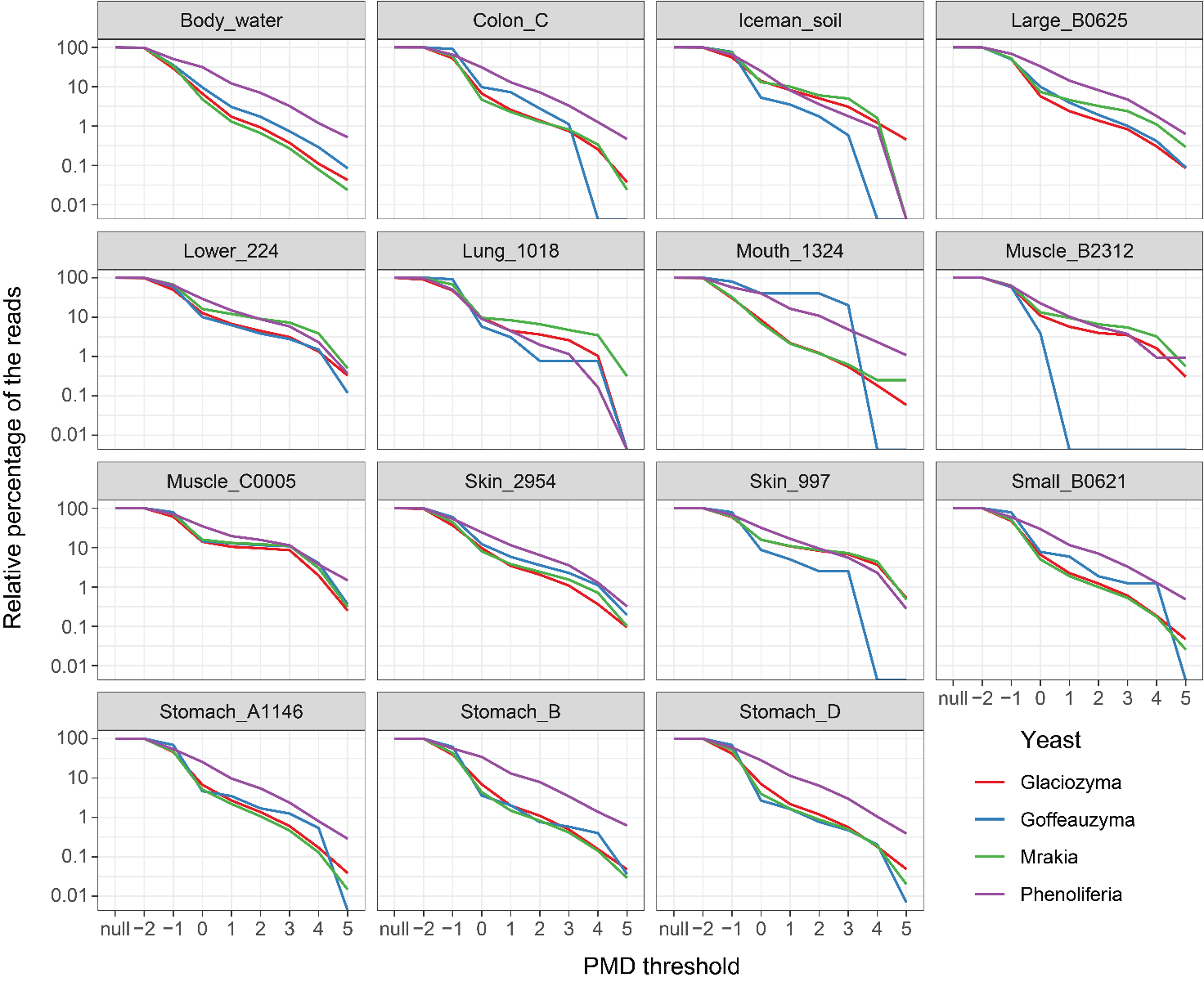
**

**Figure S6: Impact of PMD filtering on yeast read retention across Iceman samples.** The plots show the relative percentage of reads retained for Iceman yeasts (*Glaciozyma*, *Goffeauzyma*, *Mrakia*, *Phenoliferia*) as a function of increasing PMD (Post-Mortem Damage) score threshold, ranging from “null” (no filtering) to 5. Each row corresponds to a distinct Iceman sample (e.g., body water, colon, skin, stomach), with the y-axis displayed on a logarithmic scale to emphasize low-abundance changes. The samples display different patterns of decrease of reads retained after filtering, as this correlates with the biomass of living cells.

The analysis was done by mapping the short reads of all samples against the yeast draft genomes using using bowtie2 [61] and filtered for minimum mapping quality of 20 using SAMtools [52]. Then PMDtool was applied with different scores ranging from -2 to 5 [62] and ancient DNA reads proportions were counted.
